# Elevated Serum Mannose Levels as a Marker of Polycystic Ovary Syndrome

**DOI:** 10.3389/fendo.2019.00711

**Published:** 2019-10-17

**Authors:** Di Feng, Bei Shi, Fangfang Bi, Matthew Sagnelli, Xiaoli Sun, Jiao Jiao, Xiuxia Wang, Da Li

**Affiliations:** ^1^Center of Reproductive Medicine, Shengjing Hospital of China Medical University, Shenyang, China; ^2^Medical Basic Experimental Teaching Center, China Medical University, Shenyang, China; ^3^University of Connecticut School of Medicine, Farmington, CT, United States; ^4^Department of Obstetrics, Gynecology, and Reproductive Sciences, Yale School of Medicine, New Haven, CT, United States

**Keywords:** mannose, monosaccharide, PCOS (polycystic ovary syndrome), serum, insulin resistance

## Abstract

**Background:** Recent reports have highlighted the role of monosaccharide biosynthesis in the pathogenesis of polycystic ovary syndrome (PCOS), suggesting that these processes may serve as a biomarker in PCOS. Mannose is the main monosaccharide for protein glycosylation in mammals; however, the correlation between mannose and PCOS remains largely unknown.

**Materials and Methods:** A total of 132 Chinese Han women were recruited at Shengjing Hospital of China Medical University. Mannose levels were measured in serum samples collected from 71 patients with PCOS (29 lean, 42 obese) and 61 control subjects (28 lean, 33 obese). Receiver operating characteristics (ROC) curves were prepared to compare the diagnostic performance of mannose and hormonal parameters, individually or in combination. Multivariate logistic regression analysis was used to assess whether serum mannose levels were associated with PCOS after adjusting for other co-variables.

**Results:** We showed that serum mannose levels were significantly increased in PCOS patients compared with control subjects regardless of obese status, and hyperandrogenic PCOS patients had higher serum mannose levels than normo-androgenic PCOS and control subjects. In addition, serum mannose levels were significantly correlated with serum androgen levels. Mannose had an area under the curve (AUC) of 73% at a cutoff value of 225.79 ng/mL with a sensitivity of 66.2% and specificity of 73.8% for predicting PCOS. There were no differences between mannose, total testosterone, free testosterone, or dehydroepiandrosterone sulfate in the reliability of predicting PCOS using the method outlined by Hanley and McNeil. Combining mannose and total testosterone resulted in a higher AUC of 83.3%, and had moderate sensitivity (78.9%) and specificity (77%) for predicting PCOS. The positive and negative predictive values were 80% and 75.8%, respectively. Multivariate logistic regression revealed that higher serum mannose levels were strongly associated with an increased risk of PCOS (*P* = 0.016; odds ratio, 5.623; 95% confidence interval, 1.371–23.070).

**Conclusion:** Taken together, substantially elevated serum mannose levels are significantly associated with PCOS, highlighting the importance of further research into the role of mannose in the pathogenesis of PCOS.

## Introduction

Polycystic ovary syndrome (PCOS) is a heterogeneous endocrine disorder (1) and has significant and diverse reproductive and metabolic features, including obesity, insulin resistance, type 2 diabetes, and adverse cardiovascular risk profiles (2, 3). Monosaccharides are the simplest carbohydrates and serve as the main source of energy for metabolism (4). Recently, by RNA sequencing techniques, we found that monosaccharide biosynthesis is a novel pathway marker to distinguish between normal and PCOS follicles, but the relationship between monosaccharide and PCOS remains largely unknown (5).

Mannose is the predominant monosaccharide for protein glycosylation in mammals (6), a process which predominantly occurs in the liver (7). Notably, the importance of mannose in PCOS-related metabolic disorders has been increasingly recognized, for example, plasma mannose levels are significantly elevated in subjects with insulin resistance (7, 8). Mechanistically, the expression of mannose metabolism and utilization genes was markedly decreased in the livers of obese subjects with metabolic abnormalities (7, 8). In addition, studies have shown that elevated plasma mannose levels contribute to the development of several common chronic diseases, including type 2 diabetes and cardiovascular disease, rather than just being a predictive biomarker (9). Despite these advances in knowledge, the role of mannose in the pathogenesis of PCOS remains poorly understood. Thus, with the aim of elucidating the relationship between mannose levels and PCOS, the present study involved measuring and evaluating serum mannose levels from PCOS patients and control subjects.

## Materials and Methods

### Ethical Statement

This study was conducted in accordance with ethical standards and the Helsinki Declaration of 1975.

### Patients and Blood Samples

For blood sample collection, lean control patients (*n* = 28), lean patients with PCOS (*n* = 29), obese control patients (*n* = 33), and obese patients with PCOS (*n* = 42) were recruited at Shengjing Hospital of China Medical University. A female body mass index (BMI) ≥ 23 kg/m^2^ was used as the diagnostic criterion for overweightness and obesity in Asians (10). For this study, obesity refers to both overweight and obese patients. PCOS was defined according to the Rotterdam criteria (11), and the exclusion criteria are described in our previous publication (12). Briefly, these included: < 3 years since menarche; tobacco smoking; hormonal medication; pregnancy; lactation; medications (insulin-sensitizing drugs, oral contraceptives, antiandrogens, statins, aspirin, nicotinic acid, corticosteroids, and gonadotropin-releasing hormone agonists and antagonists) taken within the preceding 6 months; endocrine abnormalities such as diabetes mellitus, hyperprolactinemia, congenital adrenal hyperplasia, androgen-secreting tumor, Cushing's syndrome; and a history of any known neoplastic, infectious, or inflammatory diseases. Characteristics of the subjects are provided in Table 1 and Supplementary Table 1.

**Table 1:** Description of the study participants categorized by BMI.

	**Overweight/Obese**			**Lean**		
	**Control**	**PCOS**	***P-*value**	**Control**	**PCOS**	***P-*value**
*N*	33	42		28	29	
Age (year)	32.52 ± 4.09	31.43 ± 3.28	0.206	32.00 ± 2.72	30.72 ± 3.01	0.099
BMI (kg/m^2^)	25.57 ± 2.37	26.36 ± 1.86	0.111	20.58 ± 1.41	21.19 ± 1.62	0.133
Mannose (ng/mL)	178.32 (142.37–276.02)	280.77 (202.21–392.25)	0.001	188.16 (141.37–224.51)	245.36 (185.74–339.64)	0.004
Total testosterone (nM)	1.42 (1.01–1.91)	2.20 (1.84–2.61)	0.001	1.47 (1.20–1.90)	2.22 (1.80–2.79)	0.001
Free testosterone (nM)	0.017 (0.014–0.024)	0.026 (0.020–0.032)	0.001	0.019 (0.012–0.023)	0.030 (0.022–0.039)	0.005
SHBG (nM)	52.69 (31.86–87.22)	30.47 (20.68–50.43)	0.004	60.70 (34.45–110.50)	53.89 (31.48–80.22)	0.615
DHEAS (nM)	3190.40 (1624.55–4403.42)	4104.39 (3068.61–6063.29)	0.006	2540.31 (1708.84–4305.06)	5489.75 (3367.46–7874.31)	0.001
AMH (pmol/L)	16.07 (10.60–38.27)	60.01 (47.57–98.26)	0.001	26.28 (19.14–43.64)	62.83 (41.20–86.86)	0.001
FSH (IU/L)	7.93 ± 4.19	6.11 ± 1.59	0.012	7.42 ± 2.40	6.88 ± 2.28	0.392
LH (IU/L)	4.03 (2.95–5.02)	10.59 (6.99–14.17)	0.001	5.10 (4.00–7.12)	13.44 (9.46–21.99)	0.001
Estradiol (nM)	0.16 ± 0.07	0.24 ± 0.13	0.003	0.21 ± 0.15	0.27 ± 0.16	0.139
Prolactin (ng/mL)	10.75 ± 3.92	11.03 ± 7.22	0.841	12.50 ± 4.25	11.53 ± 5.38	0.450
TSH (μIU/mL)	1.77 (1.47–2.82)	1.96 (1.11–2.54)	0.623	1.90 (1.25–2.88)	2.10 (1.18–2.81)	0.943
FPG (mM)	5.10 ± 0.37	5.07 ± 0.38	0.778	5.00 ± 0.34	5.03 ± 0.37	0.802
FI (mIU/L)	12.50 (9.40–15.70)	15.30 (11.38–19.55)	0.046	8.80 (6.70–10.88)	8.90 (5.30–10.55)	0.565
HOMA-IR	3.15 ± 1.87	3.61 ± 1.56	0.243	2.09 ± 0.93	1.91 ± 0.86	0.439
QUICKI	0.33 ± 0.02	0.32 ± 0.02	0.120	0.35 ± 0.02	0.35 ± 0.02	0.458
TC (mM)	4.38 ± 0.77	4.86 ± 0.67	0.005	4.54 ± 0.61	4.57 ± 0.76	0.844
LDL-C (mM)	2.74 ± 0.82	3.02 ± 0.55	0.086	2.69 ± 0.56	2.65 ± 0.64	0.811
HDL-C (mM)	1.22 (1.08–1.43)	1.11 (0.93–1.31)	0.096	1.49 (1.30–1.77)	1.30 (1.13–1.49)	0.042
Triglycerides (mM)	1.27 (0.66–1.56)	1.40 (1.00–2.13)	0.123	0.87 (0.59–1.08)	0.87 (0.54–1.54)	0.376

*BMI, body mass index; SHBG, sex hormone-binding globulin; DHEAS, dehydroepiandrosterone sulfate; AMH, anti-Müllerian hormone; FSH, follicle-stimulating hormone; LH, luteinizing hormone; TSH, thyroid-stimulating hormone; FPG, fasting plasma glucose; FI, fasting serum insulin; HOMA-IR, homeostasis model assessment of insulin resistance; QUICKI, quantitative insulin-sensitivity check index; TC, total cholesterol; LDL-C, low-density lipoprotein cholesterol; HDL-C, high-density lipoprotein cholesterol. Mean ± standard deviation or median (interquartile range) are shown. The Mann–Whitney U test was used for non-normal distribution data and Student's t test was used for normal distribution data*.

To study the relationship between mannose and androgens, the study population of PCOS patients was further subdivided into a normo-androgenic and hyperandrogenic group based on serum androgen levels. Biochemical hyperandrogenism was diagnosed when serum androgen levels were as follows: total testosterone > 2.524 nmol/L, free testosterone > 0.043 nmol/L, and dehydroepiandrosterone sulfate (DHEAS) > 7649.24 nmol/L, all of which represent the 95th percentile of basal serum androgens in the control group of 61 healthy Chinese women without hirsutism or a family history of PCOS. Characteristics of the subjects in the subgroups are provided in Table 2. Blood samples were collected in the morning after an overnight fast, between the 3rd and 5th days of spontaneous menses or progestin-withdrawal bleeding. Blood samples were analyzed for lipids, along with insulin and glucose levels, by semi-automated enzymatic methods, whereas luteinizing hormone (LH), follicle-stimulating hormone (FSH), estradiol, total testosterone, prolactin, and thyroid-stimulating hormone (TSH) were assayed using a chemiluminescence analyzer.

**Table 2:** Description of the study participants categorized by androgen status.

	**Control**	**Normo-androgenic PCOS**	**Hyperandrogenic PCOS**	***P-*value**
*N*	61	37	34	
Age (year)	32.28 ± 3.51	30.97 ± 2.78	31.32 ± 3.58	0.057^a^, 0.210^b^
BMI (kg/m^2^)	23.28 ± 3.19	23.75 ± 3.52	24.79 ± 2.51	0.497^a^, 0.019^b^
Mannose (ng/mL)	183.71 (141.97–236.08)	228.90 (167.62–313.92)	292.69 (221.73–462.26)	0.010^a^, 0.001^b^
Total testosterone (nM)	1.46 (1.04–1.91)	1.91 (1.73–2.18)	2.65 (2.35–3.31)	0.001^a^^,^ ^b^
Free testosterone (nM)	0.019(0.013–0.023)	0.025 (0.017–0.029)	0.031 (0.023–0.044)	0.014^a^, 0.001^b^
SHBG (nM)	54.31 (32.67–88.72)	40.39 (25.65–56.89)	31.05 (22.01–71.98)	0.057^a^, 0.036^b^
DHEAS (nM)	2825.69 (1675.75–4320.76)	3379.62 (2518.11–4782.73)	6859.96 (4460.21–9637.74)	0.079^a^, 0.001^b^
AMH (pmol/L)	23.21 (13.60–39.02)	58.12 (44.84–87.93)	62.76 (46.96–98.85)	0.001^a^^,^^ b^
FSH (IU/L)	7.69 ± 3.46	6.23 ± 1.92	6.64 ± 1.94	0.021^a^, 0.105^b^
LH (IU/L)	4.66 (3.43–6.54)	11.32 (7.88–15.78)	12.06 (6.04–16.20)	0.001^a^^,^ ^b^
Estradiol (nM)	0.18 ± 0.12	0.27 ± 0.15	0.23 ± 0.13	0.005^a^, 0.065^b^
Prolactin (ng/mL)	11.55 ± 4.14	11.77 ± 7.58	10.64 ± 5.11	0.855^a^, 0.348^b^
TSH (μIU/mL)	1.84 (1.42–2.82)	2.23 (1.11–2.76)	1.74 (1.19–2.44)	0.626^a^, 0.235^b^
FPG (mM)	5.05 ± 0.36	5.05 ± 0.38	5.06 ± 0.38	0.926^a^, 0.925^b^
FI (mIU/L)	10.00 (8.05–14.10)	9.90 (7.55–16.91)	12.90 (8.45–15.85)	0.676^a^, 0.122^b^
HOMA-IR	2.66 ± 1.59	2.86 ± 1.69	2.98 ± 1.44	0.562^a^, 0.343^b^
QUICKI	0.34 ± 0.03	0.34 ± 0.03	0.33 ± 0.03	0.755^a^, 0.279^b^
TC (mM)	4.45 ± 0.70	4.57 ± 0.68	4.93 ± 0.72	0.415^a^, 0.002^b^
LDL-C (mM)	2.72 ± 0.71	2.83 ± 0.55	2.91 ± 0.68	0.404^a^, 0.207^b^
HDL-C (mM)	1.35 (1.13–1.64)	1.19 (0.88–1.32)	1.27 (1.05–1.48)	0.003^a^, 0.207^b^
Triglycerides (mM)	0.98 (0.64–1.38)	1.19 (0.79–1.59)	1.19 (0.84–2.33)	0.182^a^, 0.022^b^

*BMI, body mass index; SHBG, sex hormone-binding globulin; DHEAS, dehydroepiandrosterone sulfate; AMH, anti-Müllerian hormone; FSH, follicle-stimulating hormone; LH, luteinizing hormone; TSH, thyroid-stimulating hormone; FPG, fasting plasma glucose; FI, fasting serum insulin; HOMA-IR, homeostasis model assessment of insulin resistance; QUICKI, quantitative insulin-sensitivity check index; TC, total cholesterol; LDL-C, low-density lipoprotein cholesterol; HDL-C, high-density lipoprotein cholesterol. Mean ± standard deviation or median (interquartile range) are shown*.

a *Comparing the normo-androgenic PCOS patients and controls after post-hoc test*.

b *Comparing the hyperandrogenic PCOS patients and controls after post-hoc test*.

*P-values were adjusted for multiple testing using the Bonferroni method*.

This study was designed to investigate the Chinese Han population, which is the main ethnic group in China, comprising 92% of the total population. The study was approved by the Institutional Review Board at China Medical University on 28th February 2015 (reference number 2015PS108K). Written informed consent was obtained from all study participants.

### Measurement of Mannose

Serum mannose concentrations were determined by a competitive enzyme immunoassay (OKEH02595; Aviva Systems Biology, San Diego, CA, USA). Prior to the measurements, each serum sample was diluted in a 1:5 ratio with assay buffer. The assay was performed according to the manufacturer's instructions. In our experiment, the inter-assay coefficient of variation (CV) was 6.1% and the intra-assay CV was 9.6%. The range of the assay was 1.56–100 ng/mL. The antibody used was specific for human mannose and does not significantly cross-react with other relevant proteins.

### Measurement of Sex Hormone-Binding Globulin (SHBG), Free Testosterone, and DHEAS

Serum concentrations of SHBG (Human SHBG ELISA Kit; RayBiotech, Norcross, GA, USA); free testosterone (CSB-E05096h, Cusabio Biotech, Wuhan, China); and DHEAS (CSB-E05105h, Cusabio Biotech, Wuhan, China) were measured using commercial enzyme-linked immunosorbent assay kits following the manufacturer's protocol. The assay sensitivity limits for detecting SHBG, free testosterone, and DHEAS were described by the manufacturer as 1.2 pmol/L, 3.75 pg/mL, and 10 ng/mL, respectively. The intra-assay CVs were 10, 15, and 15%; and the inter-assay CVs were 12, 15, and 15%, respectively. Concentrations were determined by comparing the optical densities (450 nm) of samples with the standard curve.

### Statistical Analyses

All statistical analyses were conducted using the Statistical Packages for Social Sciences, version 22 (IBM Corp., Armonk, NY). A two independent proportions power analysis, conducted by an independent statistician via Power Analysis and Sample Size, version 11.0 (NCSS, LLC., Kaysville, Utah, USA) was used to estimate sample size. Normality of distribution of the continuous variables was assessed using the Kolmogorov–Smirnov test. Non-normally distributed variables were logarithmically transformed (log_10_) before statistical analysis. Comparisons between PCOS patients and controls were performed using the independent-sample *t* test and Mann–Whitney *U* test for normally and non-normally distributed variables, respectively. Furthermore, the one-way analysis of variance (ANOVA) with Tukey or Dunnett's *post-hoc* test (two-sided) was conducted for multi-group comparisons. The Pearson correlation coefficient was used to test the relationship between two quantitative variables. Multivariate logistic regression analysis was used to assess the strength of the association of serum mannose and PCOS. Receiver operating characteristics (ROC) curves were prepared to compare the diagnostic performance of mannose and hormonal parameters, either individually or in combination. The area under the ROC curve (AUC) with 95% confidence interval (CI), sensitivity, specificity, positive predictive value (PPV), and negative predictive value (NPV) for the diagnosis of PCOS were calculated. The Youden index was calculated to determine the optimal cutoff point. Descriptive results of study participants are expressed as means ± standard error (SE) or median (interquartile range). Bonferroni tests were performed to adjust for multiple testing. All tests were two-sided, and a *P* value of < 0.05 was considered to convey statistically significant differences.

## Results

### PCOS Patients Exhibit High Serum Mannose Levels

**Figure 1:**
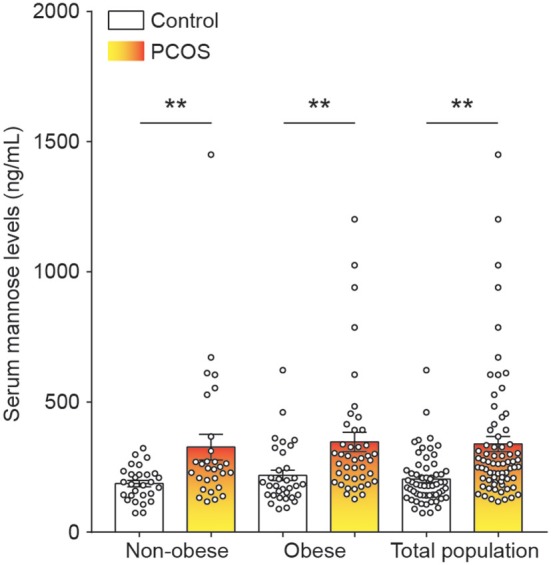
Mannose levels in serum of control subjects and PCOS patients. Differences in serum mannose levels between non-obese and obese control subjects and PCOS patients. Bar graphs show the mean ± standard error. **P < 0.01 vs. control. PCOS, polycystic ovary syndrome.

To systematically study the relationship between serum mannose levels and PCOS in the context of BMI, the study population of control subjects and PCOS patients was divided into obese and non-obese subgroups. As shown in Figure 1 and Table 1, serum mannose levels were significantly higher in PCOS patients than in the control subjects, regardless of obese status. Accordingly, serum mannose levels were greater in PCOS patients than in control subjects when the non-obese and obese subgroups were combined (Figure 1 and Supplementary Table 1).

### Serum Mannose Levels Showed a Positive Relationship and Similar Diagnostic Performance to Androgens in PCOS

**Figure 2:**
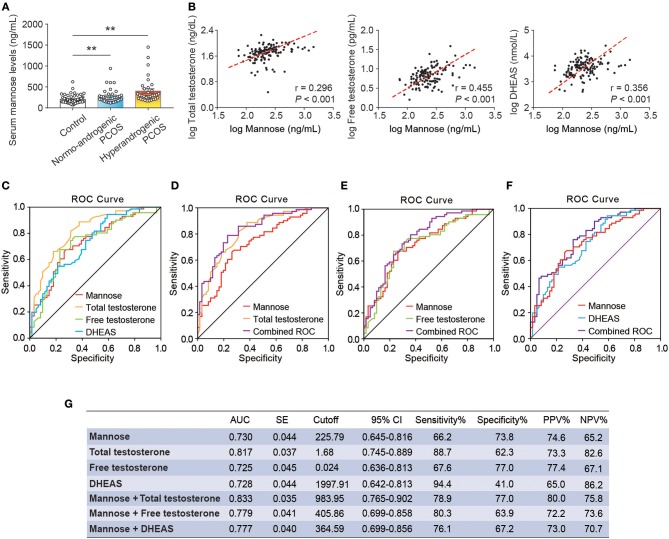
Correlation and ROC analysis between serum mannose and androgens. **(A)** Differences in serum mannose levels in control subjects, normo-androgenic PCOS patients, and hyperandrogenic PCOS patients. **(B)** Correlation between serum total testosterone and free testosterone, and DHEAS and mannose levels, respectively. Both parameters were log-transformed for the plot. **(C–G)** Diagnostic potential of serum mannose, total testosterone, free testosterone, and DHEAS estimated using ROC analysis. ROC curve analysis of the individual androgens, and combining the individual androgens with mannose, respectively. AUC, cutoff value, sensitivity, and specificity are indicated. Bar graphs show the mean ± standard error (SE). **P < 0.01 vs. control. PCOS, polycystic ovary syndrome; DHEAS, dehydroepiandrosterone sulfate; ROC, receiver operating characteristic; AUC, the area under the curve; CI, confidence interval; PPV, positive predictive value; NPV, negative predictive value.

As stated, serum mannose levels were markedly elevated in PCOS patients. Therefore, using ROC curves, we compared the performance of mannose levels with androgens as diagnostic indicators of PCOS. As shown in Figure 2A and Table 2, serum mannose levels were higher in normo-androgenic PCOS patients than in control subjects. In addition, mannose levels were also elevated in the hyperandrogenic PCOS patients compared to normo-androgenic PCOS patients. Correlation analyses were used to examine the relationship between serum mannose levels and the major androgens. Notably, serum mannose levels were correlated with total testosterone and DHEAS levels, and exhibited a stronger association with free testosterone (Figure 2B). The diagnostic performance of mannose was evaluated using ROC curves and compared with total testosterone, free testosterone, and DHEAS. As shown in Figures 2C,G, mannose had an AUC of 73% at a cutoff value of 225.79 ng/mL with a sensitivity of 66.2% and specificity of 73.8% for predicting PCOS. The PPV and NPV were 74.6% and 65.2%, respectively. There were no differences between mannose, total testosterone, free testosterone, and DHEAS in the reliability of predicting PCOS using the method outlined by Hanley and McNeil (13). However, when mannose and total testosterone levels were combined, there was a higher AUC of 83.3%, with moderate sensitivity (78.9%) and specificity (77%) for predicting PCOS (Figures 2D,G). The PPV and NPV were 80% and 75.8%, respectively. The combination of mannose and free testosterone resulted in an AUC of 77.9%, with moderate diagnostic sensitivity (80.3%) and poor diagnostic specificity (63.9%) for PCOS (Figures 2E,G). Combining mannose and DHEAS resulted in an AUC of 77.7%, with moderate diagnostic sensitivity (76.1%) and poor diagnostic specificity (67.2%) for predicting PCOS (Figures 2F,G). Notably, multivariate logistic regression revealed that elevated serum mannose levels were strongly associated with a high risk of PCOS (*P* = 0.016; odds ratio, 5.623; 95% confidence interval, 1.371–23.070; Table 3).

**Table 3:** Multivariate analysis with the following variables in the model: mannose, androgens, AMH, FSH, LH, FI, and BMI.

**Variable**	**OR**	**95%CI**	***P-*value**
Total testosterone (nM)	7.750	2.050–29.296	0.003
Free testosterone (nM)	0.838	0.159–4.419	0.835
DHEAS (nM)	4.086	0.953–17.527	0.058
AMH (pmol/L)	1.009	0.996–1.022	0.175
FSH (IU/L)	0.945	0.788–1.132	0.539
LH (IU/L)	0.983	0.924–1.045	0.577
FI (mIU/L)	0.825	0.367–1.856	0.642
BMI (kg/m^2^)	0.932	0.407–2.137	0.868
Mannose (ng/mL)	5.623	1.371-23.070	0.016

*DHEAS, dehydroepiandrosterone sulfate; AMH, anti-Müllerian hormone; FSH, follicle-stimulating hormone; LH, luteinizing hormone; FI, fasting serum insulin; BMI, body mass index; OR, odds ratio; CI, confidence interval*.

*P-value was calculated by multivariate logistic regression*.

## Discussion

In this study, we reported for the first time that serum mannose levels are significantly increased in PCOS patients, and that hyperandrogenic PCOS patients had higher serum mannose levels compared to normo-androgenic PCOS patients and control subjects. In addition, serum mannose levels were significantly correlated with serum androgen levels. These observations suggest a link between mannose and androgen metabolism. Notably, one previous report highlighted the function of the mannose receptor (ManR, Mrc1) in androgen metabolism, suggesting that the half-life for LH clearance was increased in Mrc1^−/−^ mice, and that LH levels were elevated because LH was no longer cleared by the ManR, resulting in higher levels of testosterone (14). Conversely, Kowalska et al. observed that the concentration of serum mannan-binding lectin (MBL) was decreased in PCOS, but they did not find any association between MBL and markers of hyperandrogenism (15). Despite these reports, the direct relationship between mannose and androgens remains largely unknown. These preliminary results open new avenues toward improving our understanding of the biological role of mannose in PCOS-related androgen disorders. Apart from androgens, it is well-known that elevated serum LH and anti-Müllerian hormone (AMH) levels are common features in PCOS (11, 16, 17), and positive correlations between serum concentrations of LH, AMH, and testosterone in PCOS have been widely reported (16). Therefore, we further examined the relationship between serum mannose, LH/FSH, and AMH levels, but no correlations were observed between serum mannose and LH/FSH or AMH levels (data not shown). The present study assessed whether serum mannose levels remained associated with PCOS subsequent to adjusting for other co-variables. Parameters that are commonly abnormal in PCOS, such as serum androgens, AMH, FSH, LH, fasting serum insulin (FI), and BMI were used as variables in a multivariate logistic regression model. It is important to note that, similar to total testosterone, serum mannose can be considered as an independent predictive factor for PCOS.

Although there is an inadequate understanding of the etiology of PCOS, insulin resistance is a relatively common feature of the disease (18) and the pathophysiology of PCOS involves alterations in insulin action in a variety of target tissues (19). Recent reports have identified mannose as having one of the strongest positive associations with insulin resistance among plasma metabolites (7–9). In agreement with these findings, we observed that elevated serum mannose levels are accompanied by increased FI levels in the PCOS patients in the obese subgroup. Interestingly, Dahan et al. reported that FI can be considered as a simple and accurate surrogate predictor of insulin resistance in PCOS patients and control subjects (20). Taken together, these studies suggest that mannose-related insulin resistance may have a role in the pathogenesis of PCOS, especially in obese PCOS patients.

The results of the present study reveal differences in serum mannose levels between control subjects and PCOS patients; however, it remains unclear whether mannose contributes directly to the pathogenesis of PCOS or are is just a biomarker of this process. Together with our previous study (5), the present results highlight the importance of further investigation into the role of mannose in the biological mechanisms underlying the pathogenesis of PCOS.

## Data Availability Statement

The datasets generated for this study are available on request to the corresponding author.

## Ethics Statement

All subjects signed a written informed consent before participating in the study which abides by the Declaration of Helsinki and was approved by the Ethical Review Board at China Medical University.

## Author Contributions

DL and XW conceived and designed the study. DF, BS, DL, FB, JJ, MS, and XS performed data acquisition and interpretation. DL, XW, BS, and DF wrote the paper. All authors approved the final manuscript.

### Conflict of Interest

The authors declare that the research was conducted in the absence of any commercial or financial relationships that could be construed as a potential conflict of interest.

## References

[1] Goodarzi MO Dumesic DA Chazenbalk G Azziz R. Polycystic ovary syndrome: etiology, pathogenesis and diagnosis. Nat Rev Endocrinol. (2011) 7:219–31. 10.1038/nrendo.2010.217 21263450

[2] Chan JL Kar S Vanky E Morin-Papunen L Piltonen T Puurunen J. Racial and ethnic differences in the prevalence of metabolic syndrome and its components of metabolic syndrome in women with polycystic ovary syndrome: a regional cross-sectional study. Am J Obstet Gynecol. (2017) 217:189.e1–8. 10.1016/j.ajog.2017.04.007 28400308

[3] Teede H Deeks A Moran L. Polycystic ovary syndrome: a complex condition with psychological, reproductive and metabolic manifestations that impacts on health across the lifespan. BMC Med. (2010) 8:41. 10.1186/1741-7015-8-41 20591140 PMC2909929

[4] Dashty M. A quick look at biochemistry: carbohydrate metabolism. Clin Biochem. (2013) 46:1339–52. 10.1016/j.clinbiochem.2013.04.027 23680095

[5] Jiao J Shi B Wang T Fang Y Cao T Zhou Y. Characterization of long non-coding RNA and messenger RNA profiles in follicular fluid from mature and immature ovarian follicles of healthy women and women with polycystic ovary syndrome. Hum Reprod. (2018) 33:1735–48. 10.1093/humrep/dey255 30052945

[6] Sharma V Smolin J Nayak J Ayala JE Scott DA Peterson SN. Mannose alters gut microbiome, prevents diet-induced obesity, and improves host metabolism. Cell Rep. (2018) 24:3087–98. 10.1016/j.celrep.2018.08.064 30231992 PMC6207501

[7] Lee S Zhang C Kilicarslan M Piening BD Bjornson E Hallstrom BM. Integrated network analysis reveals an association between plasma mannose levels and insulin resistance. Cell Metab. (2016) 24:172–84. 10.1016/j.cmet.2016.05.026 27345421 PMC6666317

[8] Holmes D. Biomarkers: Mannose levels predict insulin resistance. Nat Rev Endocrinol. (2016) 12:496. 10.1038/nrendo.2016.119 27448056

[9] Mardinoglu A Stancakova A Lotta LA Kuusisto J Boren J Bluher M. Plasma mannose levels are associated with incident type 2 diabetes and cardiovascular disease. Cell Metab. (2017) 26:281–3. 10.1016/j.cmet.2017.07.006 28768165

[10] World Health Organization International Obesity Task Force The Asian-Pacific Perspective: Redefining Obesity and Its Treatment. (2000). WHO Western Pacific Region, Geneva, Switzerland.

[11] Rotterdam EA-SPCWG Revised 2003 consensus on diagnostic criteria and long-term health risks related to polycystic ovary syndrome. Fertil Steril. (2004) 81:19–25. 10.1016/j.fertnstert.2003.10.004 14711538

[12] Li D Liu HX Fang YY Huo JN Wu QJ Wang TR. Hyperhomocysteinemia in polycystic ovary syndrome: decreased betaine-homocysteine methyltransferase and cystathionine β-synthase-mediated homocysteine metabolism. Reprod Biomed Online. (2018) 37:234–41. 10.1016/j.rbmo.2018.05.008 29804940

[13] Hanley JA McNeil BJ. A method of comparing the areas under receiver operating characteristic curves derived from the same cases. Radiology. (1983) 148:839–43. 10.1148/radiology.148.3.6878708 6878708

[14] Mi Y Coonce M Fiete D Steirer L Dveksler G Townsend RR. Functional consequences of mannose and asialoglycoprotein receptor ablation. J Biol Chem. (2016) 291:18700–17. 10.1074/jbc.M116.738948 27405760 PMC5009246

[15] Kowalska I Fernandez-Real JM Straczkowski M Kozlowska A Adamska A Ortega F. Insulin resistance is associated with decreased circulating mannan-binding lectin concentrations in women with polycystic ovary syndrome. Diabetes Care. (2008) 31:e20. 10.2337/dc07-1872 18375421

[16] Homburg R Ray A Bhide P Gudi A Shah A Timms P. The relationship of serum anti-Mullerian hormone with polycystic ovarian morphology and polycystic ovary syndrome: a prospective cohort study. Hum Reprod. (2013) 28:1077–83. 10.1093/humrep/det015 23377771

[17] Iliodromiti S Kelsey TW Anderson RA Nelson SM. Can anti-Mullerian hormone predict the diagnosis of polycystic ovary syndrome? A systematic review and meta-analysis of extracted data. J Clin Endocrinol Metab. (2013) 98:3332–40. 10.1210/jc.2013-1393 23775353

[18] Amato MC Vesco R Vigneri E Ciresi A Giordano C. Hyperinsulinism and polycystic ovary syndrome (PCOS): role of insulin clearance. J Endocrinol Invest. (2015) 38:1319–26. 10.1007/s40618-015-0372-x 26294351

[19] Dumesic DA Oberfield SE Stener-Victorin E Marshall JC Laven JS Legro RS. Scientific statement on the diagnostic criteria, epidemiology, pathophysiology, and molecular genetics of polycystic ovary syndrome. Endocr Rev. (2015) 36:487–525. 10.1210/er.2015-1018 26426951 PMC4591526

[20] Dahan MH Abbasi F Reaven G. Relationship between surrogate estimates and direct measurement of insulin resistance in women with polycystic ovary syndrome. J Endocrinol Invest. (2019) 42:987–93. 10.1007/s40618-019-01014-9 30701438 PMC6639126

