# Supplementary material for: Elevated Serum Mannose Levels as a Marker of Polycystic Ovary Syndrome
Source: Front Endocrinol (Lausanne). 2019 Oct 17;10:711. doi: 10.3389/fendo.2019.00711 (PMC6811522; doi:10.3389/fendo.2019.00711)
Supplement: Supplementary file 1 [file Table_1.DOCX]

**Supplementary Table 1**. Description of the study participants.

|  | Control | PCOS | *P-*value |
| --- | --- | --- | --- |
| N | 61 | 71 |  |
| Age (year) | 32.28 ± 3.51 | 31.14 ± 3.17 | 0.053 |
| BMI (kg/m^2^) | 23.28 ± 3.19 | 24.25 ± 3.10 | 0.080 |
| **Mannose (ng/mL)** | **183.71 (141.97-236.08)** | **262.99 (200.08-367.25)** | **0.001** |
| Total testosterone (nM) | 1.46 (1.04-1.91) | 2.22 (1.84-2.63) | 0.001 |
| Free testosterone (nM) | 0.019 (0.013-0.023) | 0.026 (0.021-0.037) | 0.001 |
| SHBG (nM) | 54.31 (32.67-88.73) | 36.24 (24.28-66.42) | 0.016 |
| DHEAS (nM) | 2825.69 (1675.75-4320.76) | 4468.54 (3250.43-7082.76) | 0.001 |
| AMH (pmol/L) | 23.21 (13.60-39.02) | 62.62 (46.27-95.68) | 0.001 |
| FSH (IU/L) | 7.69 ± 3.46 | 6.43 ± 1.93 | 0.009 |
| LH (IU/L) | 4.66 (3.43-6.54) | 11.65 (7.73-16.09) | 0.001 |
| Estradiol (nM) | 0.18 ± 0.12 | 0.25 ± 0.14 | 0.003 |
| Prolactin (ng/mL) | 11.55 ± 4.14 | 11.23 ± 6.49 | 0.739 |
| TSH (µIU/mL) | 1.84 (1.42-2.82) | 1.97 (1.16-2.64) | 0.707 |
| FPG (mM) | 5.05 ± 0.36 | 5.05 ± 0.37 | 0.998 |
| FI (mIU/L) | 10.00 (8.05-14.10) | 11.50 (8.30-16.00) | 0.243 |
| HOMA-IR | 2.66 ± 1.59 | 2.92 ± 1.56 | 0.359 |
| QUICKI | 0.34 ± 0.03 | 0.33 ± 0.03 | 0.415 |
| TC (mM) | 4.45 ± 0.70 | 4.74 ± 0.72 | 0.020 |
| LDL-C (mM) | 2.72 ± 0.71 | 2.87 ± 0.61 | 0.193 |
| HDL-C (mM) | 1.35 (1.13-1.64) | 1.22 (0.97-1.40) | 0.010 |
| Triglycerides (mM) | 0.98 (0.64-1.38) | 1.19 (0.83-1.90) | 0.029 |

**Abbreviations:** BMI, body mass index; SHBG, sex hormone-binding globulin; DHEAS, dehydroepiandrosterone sulfate; AMH, anti-Müllerian hormone; FSH, follicle-stimulating hormone; LH, luteinizing hormone; TSH, thyroid-stimulating hormone; FPG, fasting plasma glucose; FI, fasting serum insulin; HOMA-IR, homeostasis model assessment of insulin resistance; QUICKI, quantitative insulin-sensitivity check index; TC, total cholesterol; LDL-C, low-density lipoprotein cholesterol; HDL-C, high-density lipoprotein cholesterol. Mean ± standard deviation or median (interquartile range) are shown. The Mann–Whitney *U* test was used for non-normal distribution data and Student’s *t* test was used for normal distribution data.
